# Supplementary figures and images for: Computational image analysis reveals the structural complexity of Toxoplasma gondii tissue cysts
Source: PLoS One. 2020 Aug 18;15(8):e0234169. doi: 10.1371/journal.pone.0234169 (PMC7444489; doi:10.1371/journal.pone.0234169)

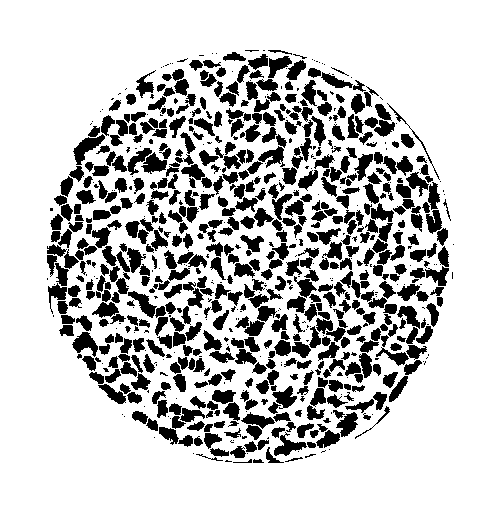

Supplement: S3 Data — (ZIP) [file pone.0234169.s003.zip › Watershed segmentation/BGD1/BGD1-01.tif]

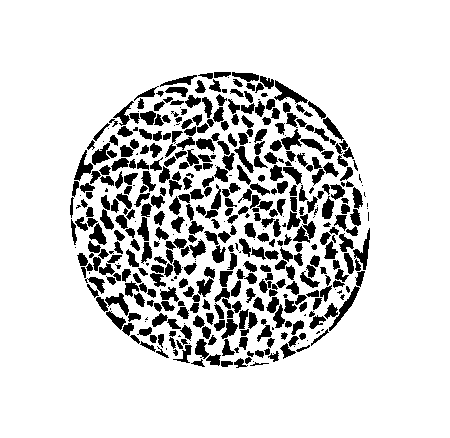

Supplement: S3 Data — (ZIP) [file pone.0234169.s003.zip › Watershed segmentation/BGD1/BGD1-02.tif]

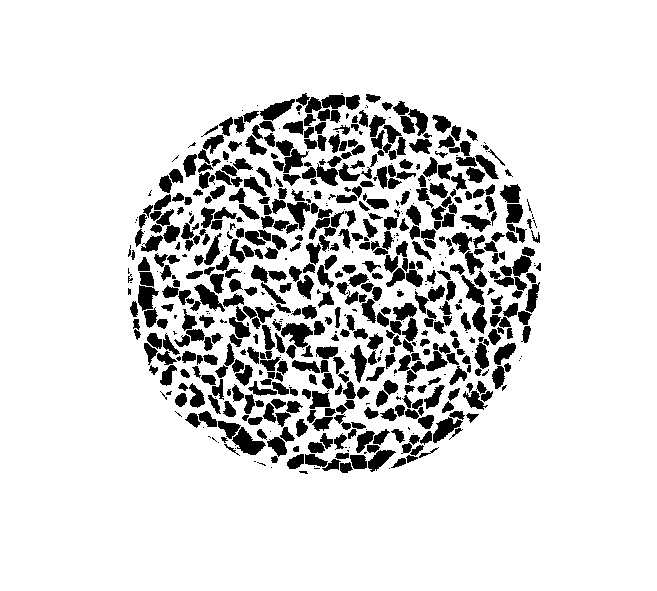

Supplement: S3 Data — (ZIP) [file pone.0234169.s003.zip › Watershed segmentation/BGD1/BGD1-03.tif]

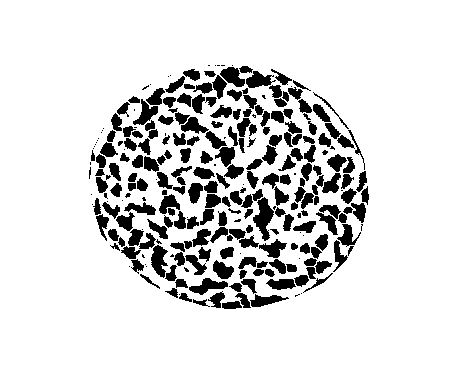

Supplement: S3 Data — (ZIP) [file pone.0234169.s003.zip › Watershed segmentation/BGD1/BGD1-04.tif]

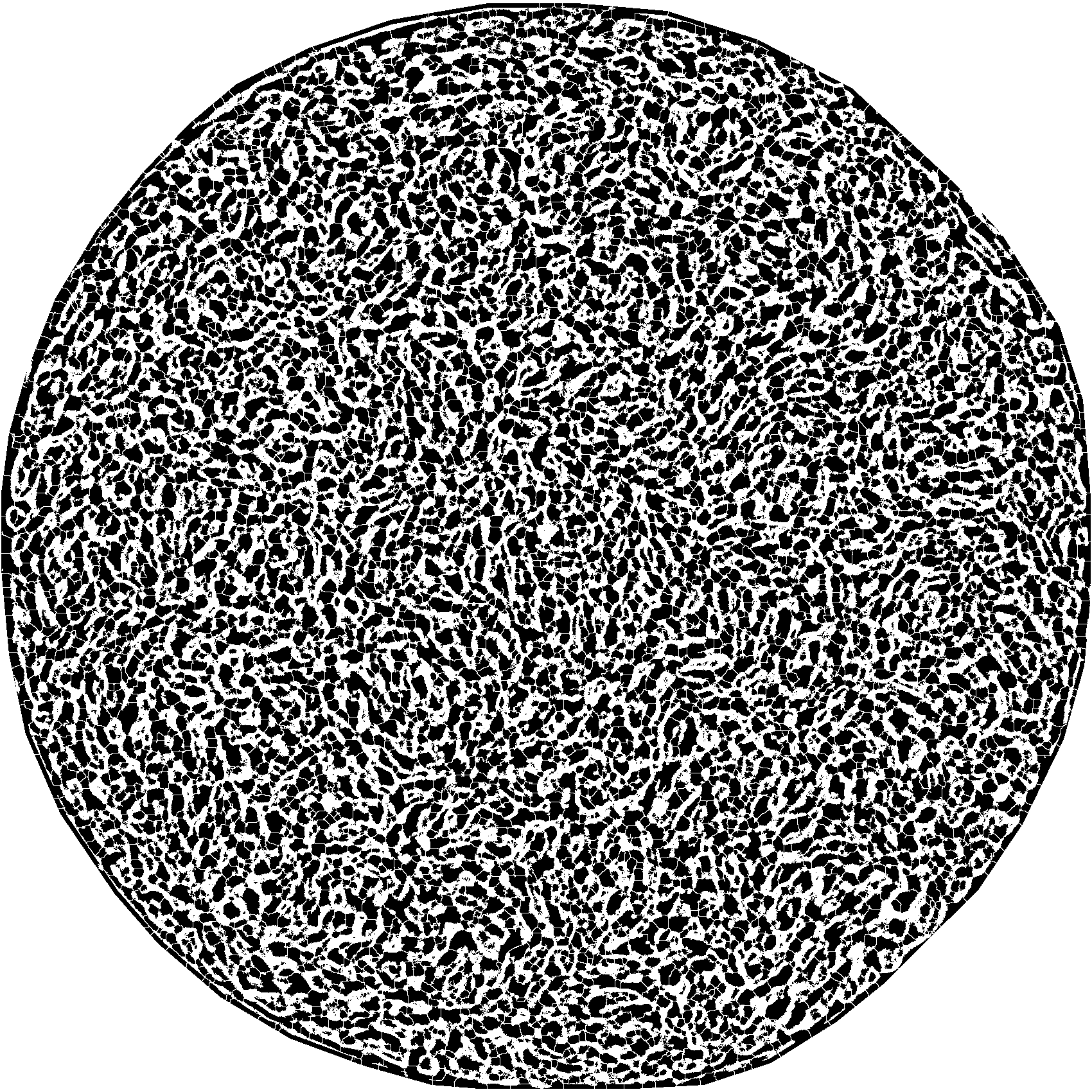

Supplement: S3 Data — (ZIP) [file pone.0234169.s003.zip › Watershed segmentation/BGD1/BGD1-05.tif]

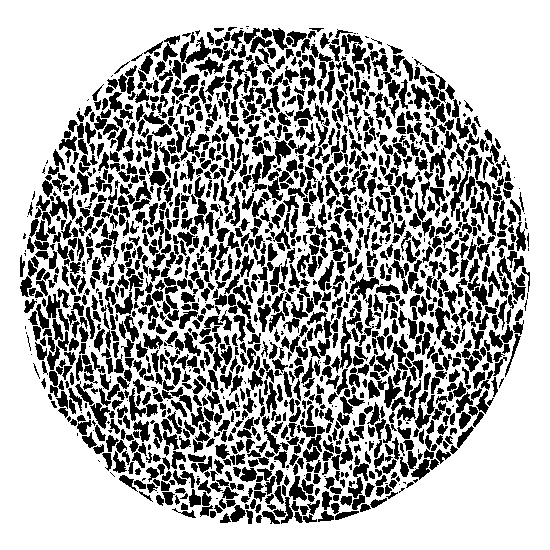

Supplement: S3 Data — (ZIP) [file pone.0234169.s003.zip › Watershed segmentation/BGD1/BGD1-06.tif]

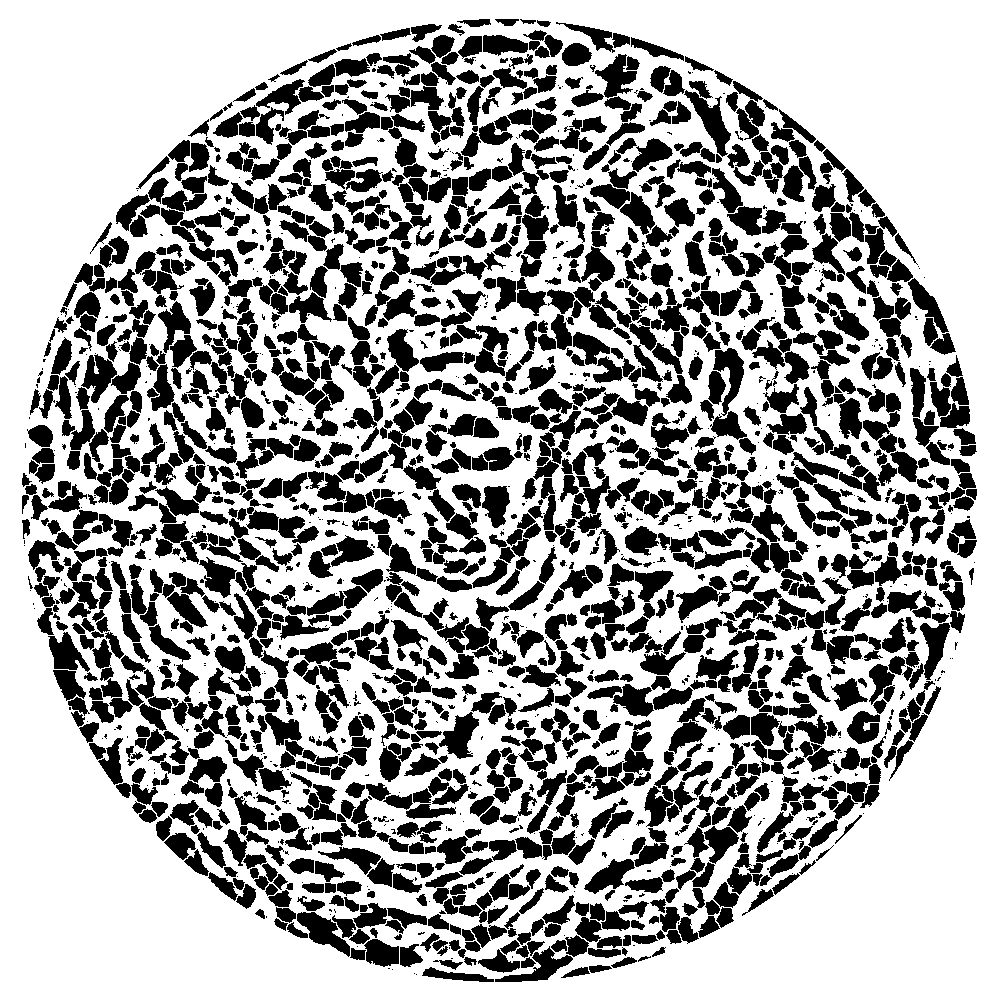

Supplement: S3 Data — (ZIP) [file pone.0234169.s003.zip › Watershed segmentation/BGD14/BGD14-01.tif]

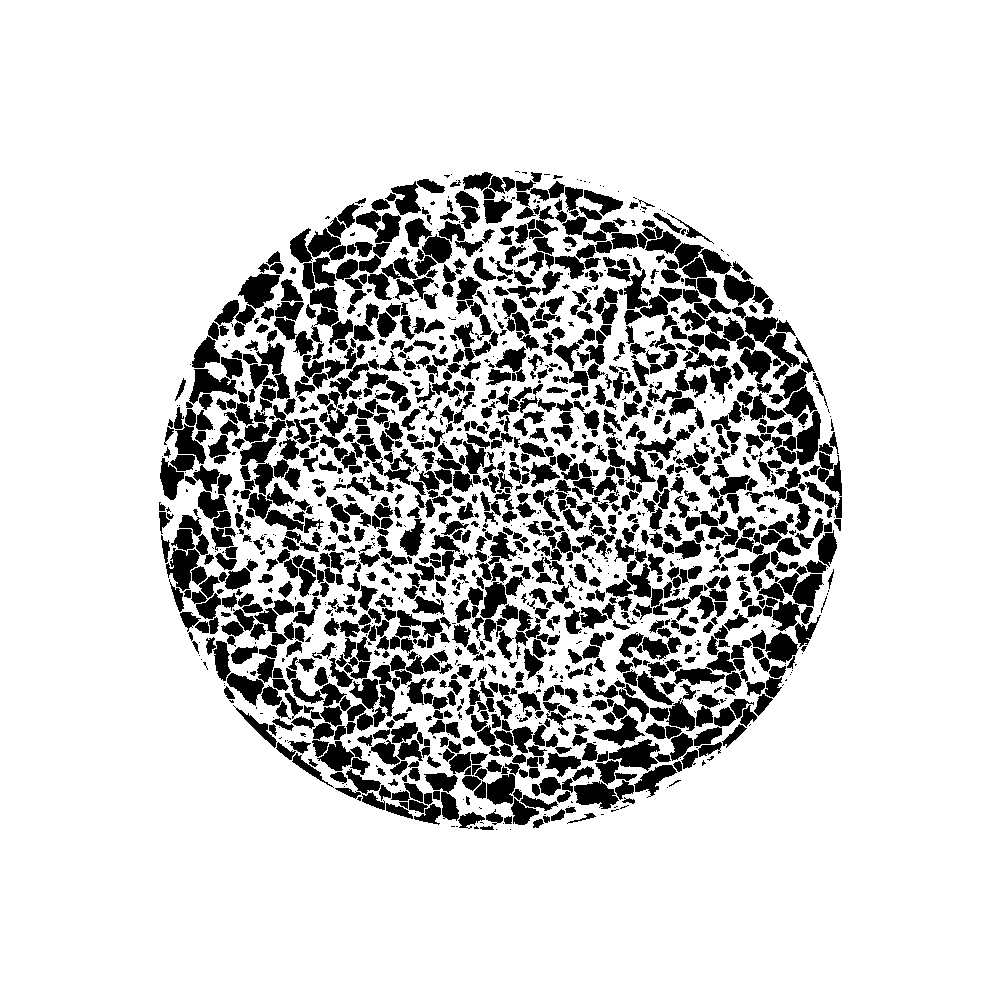

Supplement: S3 Data — (ZIP) [file pone.0234169.s003.zip › Watershed segmentation/BGD14/BGD14-02.tif]

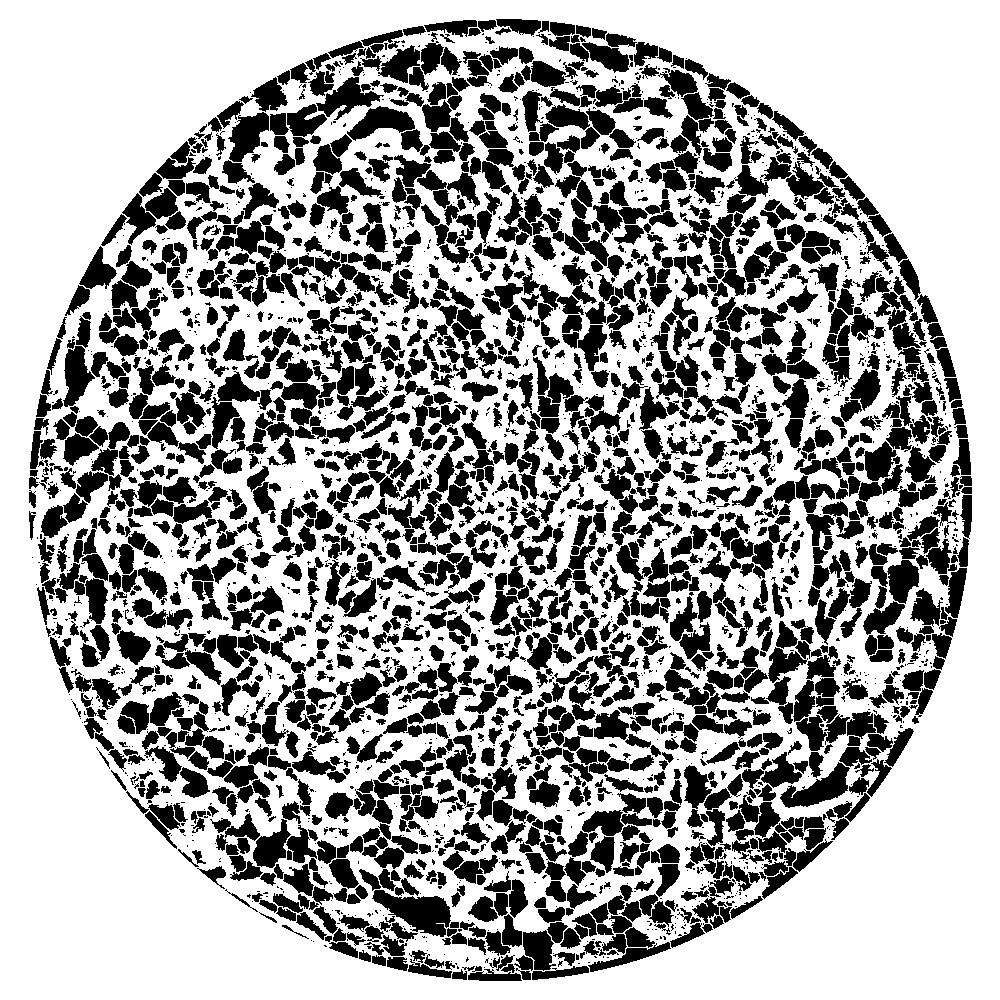

Supplement: S3 Data — (ZIP) [file pone.0234169.s003.zip › Watershed segmentation/BGD14/BGD14-03.tif]

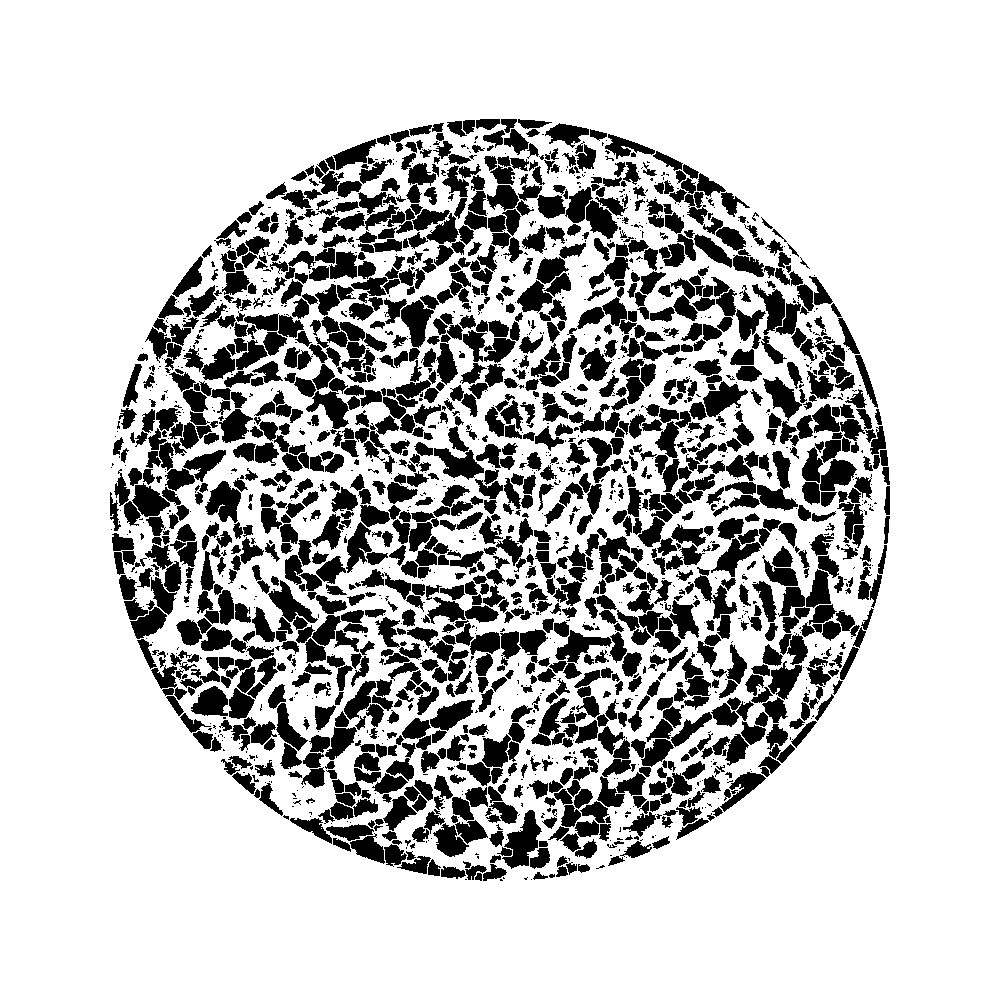

Supplement: S3 Data — (ZIP) [file pone.0234169.s003.zip › Watershed segmentation/BGD14/BGD14-04.tif]

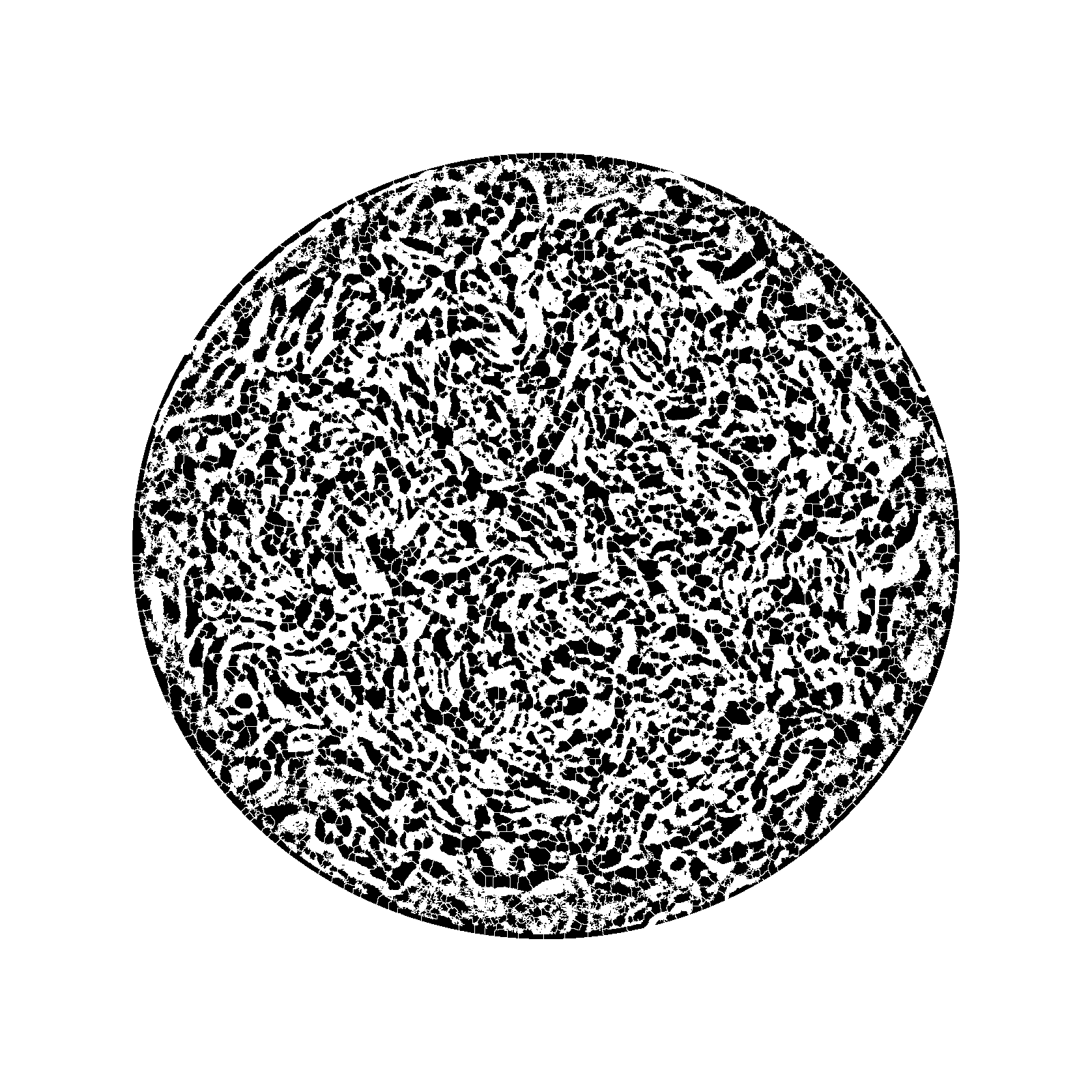

Supplement: S3 Data — (ZIP) [file pone.0234169.s003.zip › Watershed segmentation/BGD14/BGD14-05.tif]

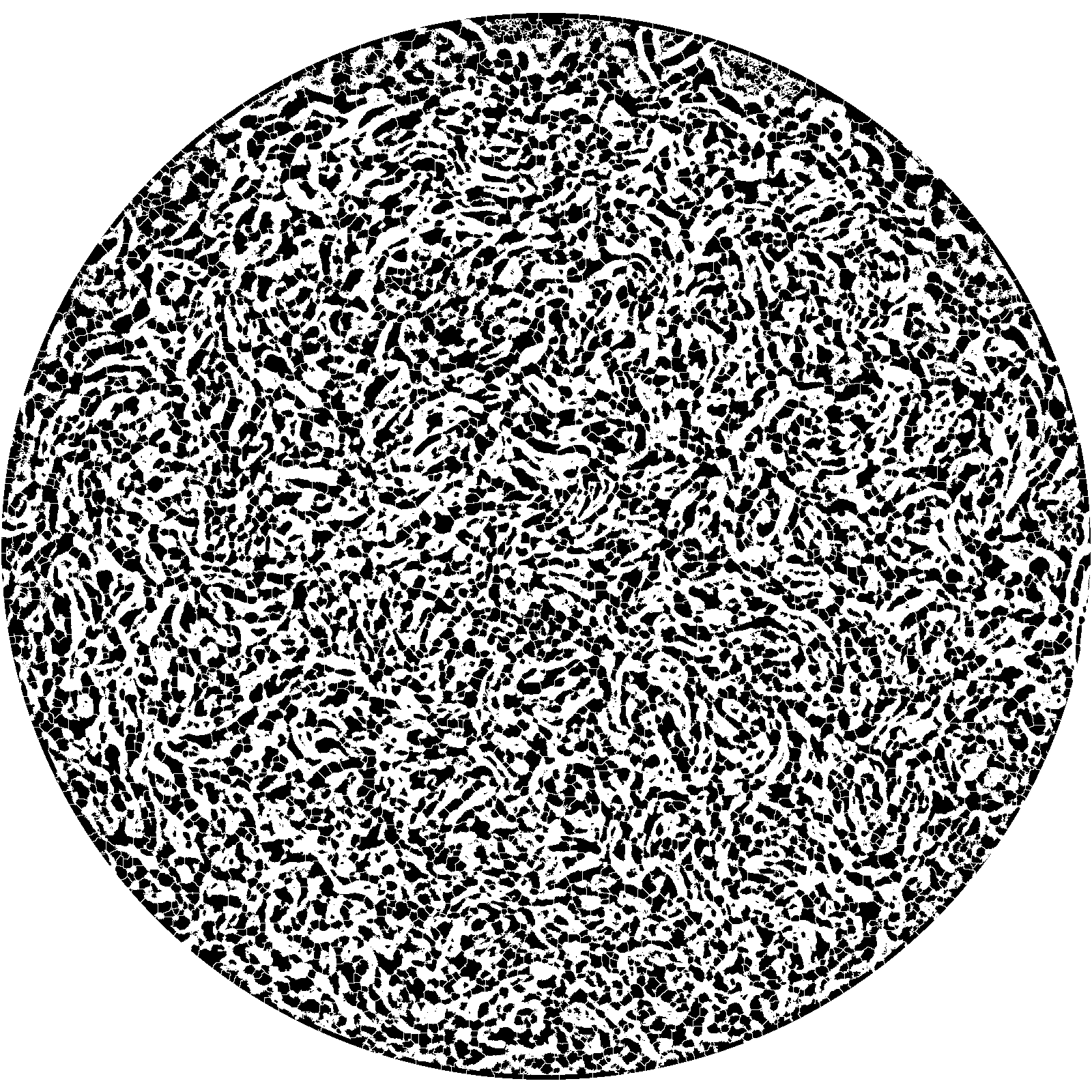

Supplement: S3 Data — (ZIP) [file pone.0234169.s003.zip › Watershed segmentation/BGD14/BGD14-06.tif]

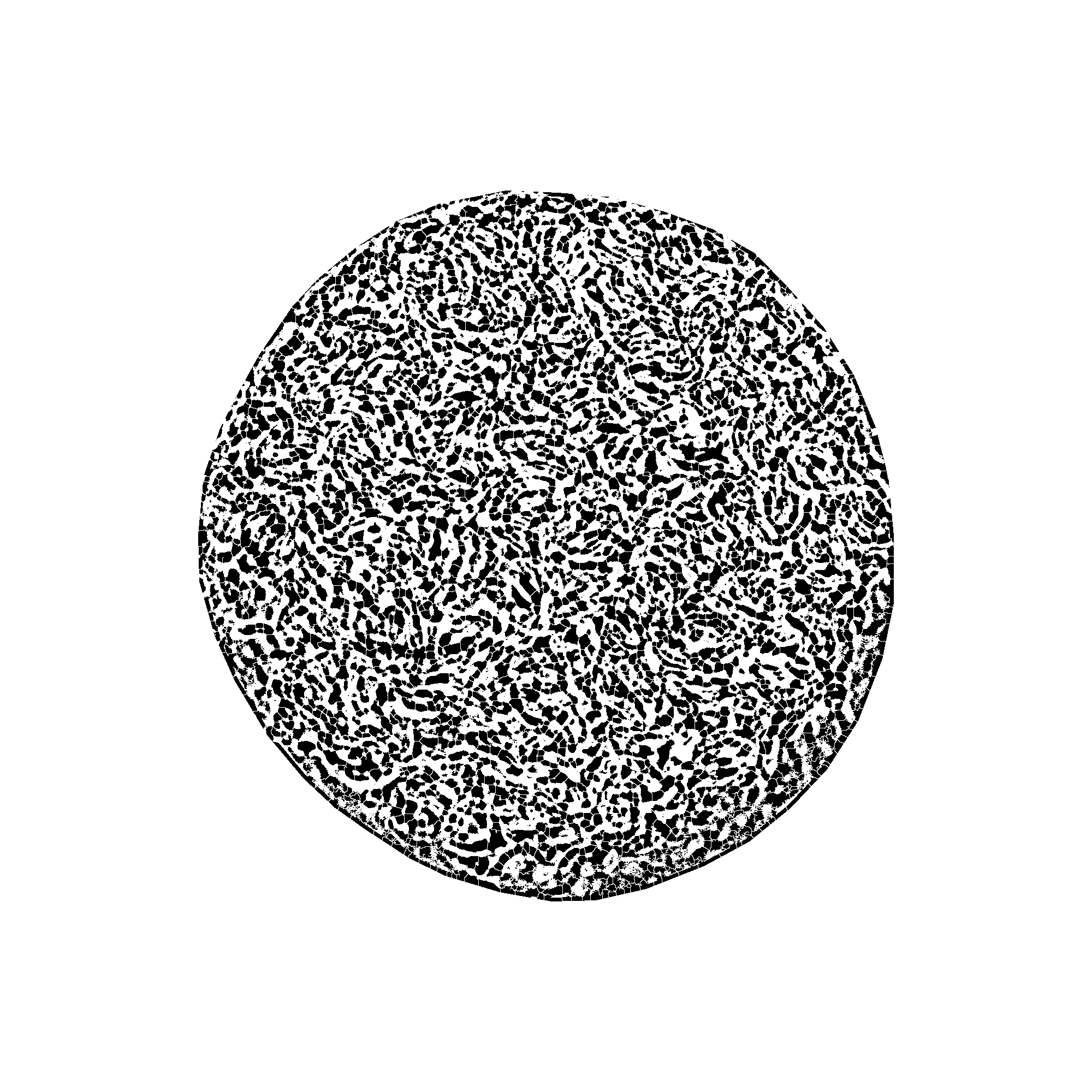

Supplement: S3 Data — (ZIP) [file pone.0234169.s003.zip › Watershed segmentation/BGD26/BGD26-01.tif]

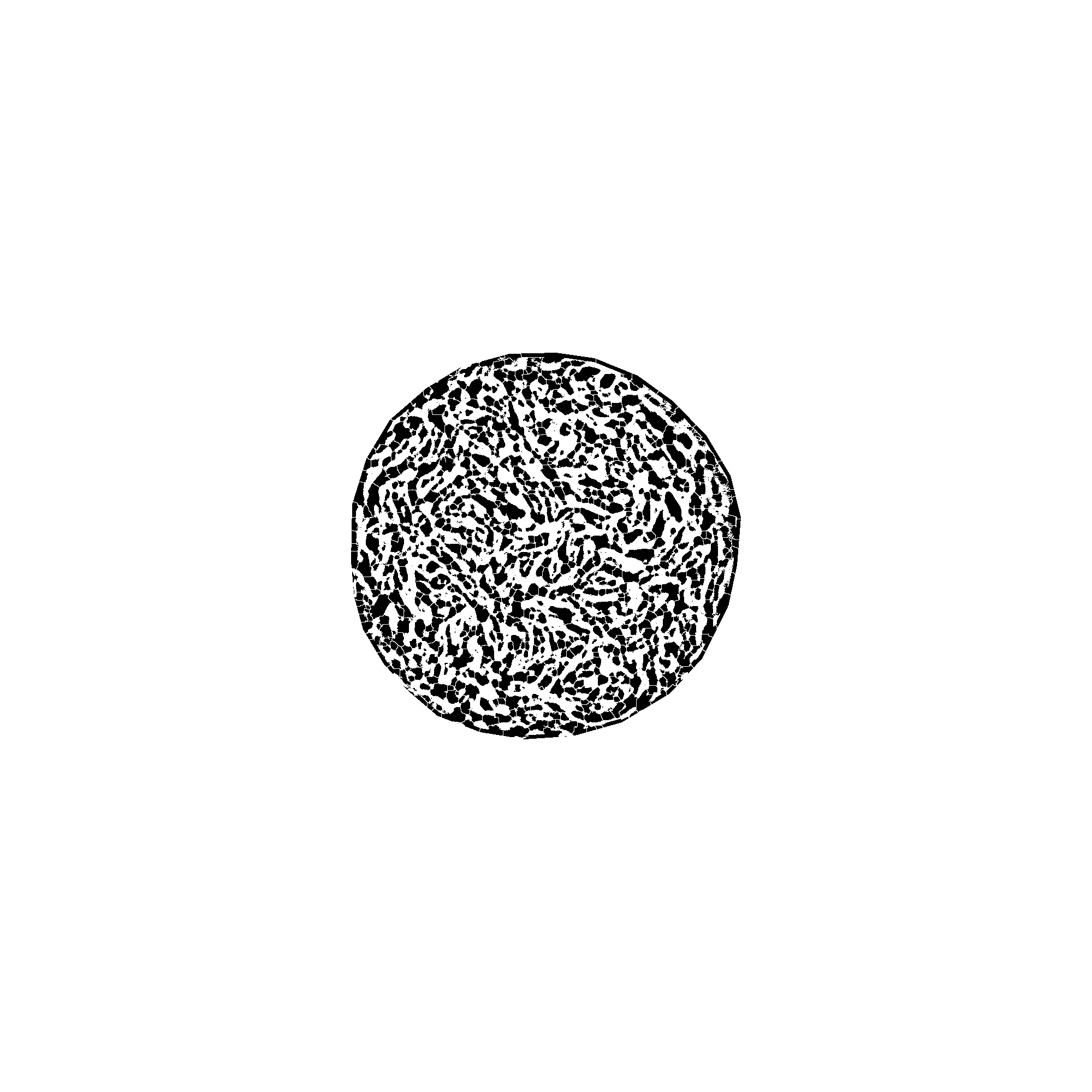

Supplement: S3 Data — (ZIP) [file pone.0234169.s003.zip › Watershed segmentation/BGD26/BGD26-02.tif]

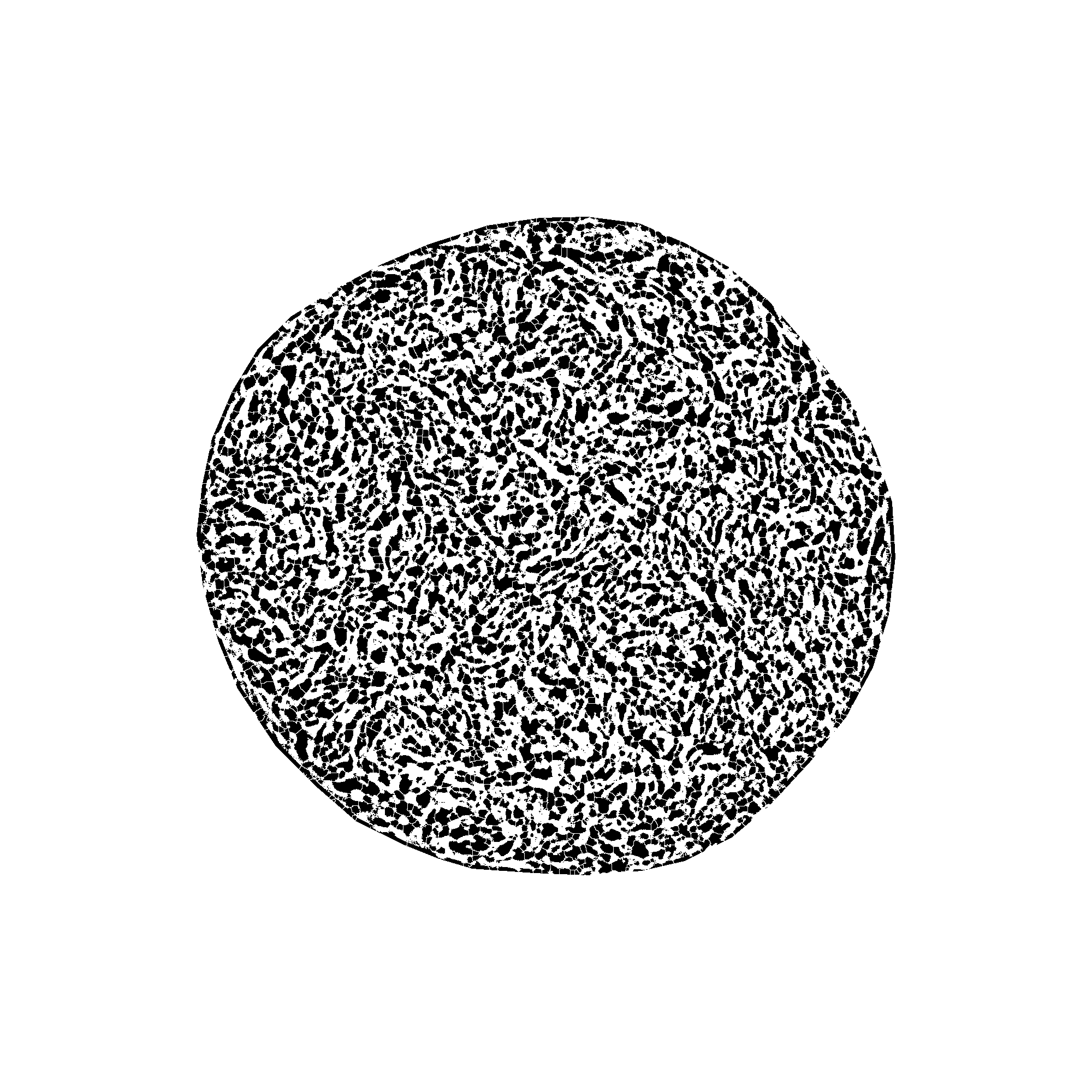

Supplement: S3 Data — (ZIP) [file pone.0234169.s003.zip › Watershed segmentation/BGD26/BGD26-03.tif]

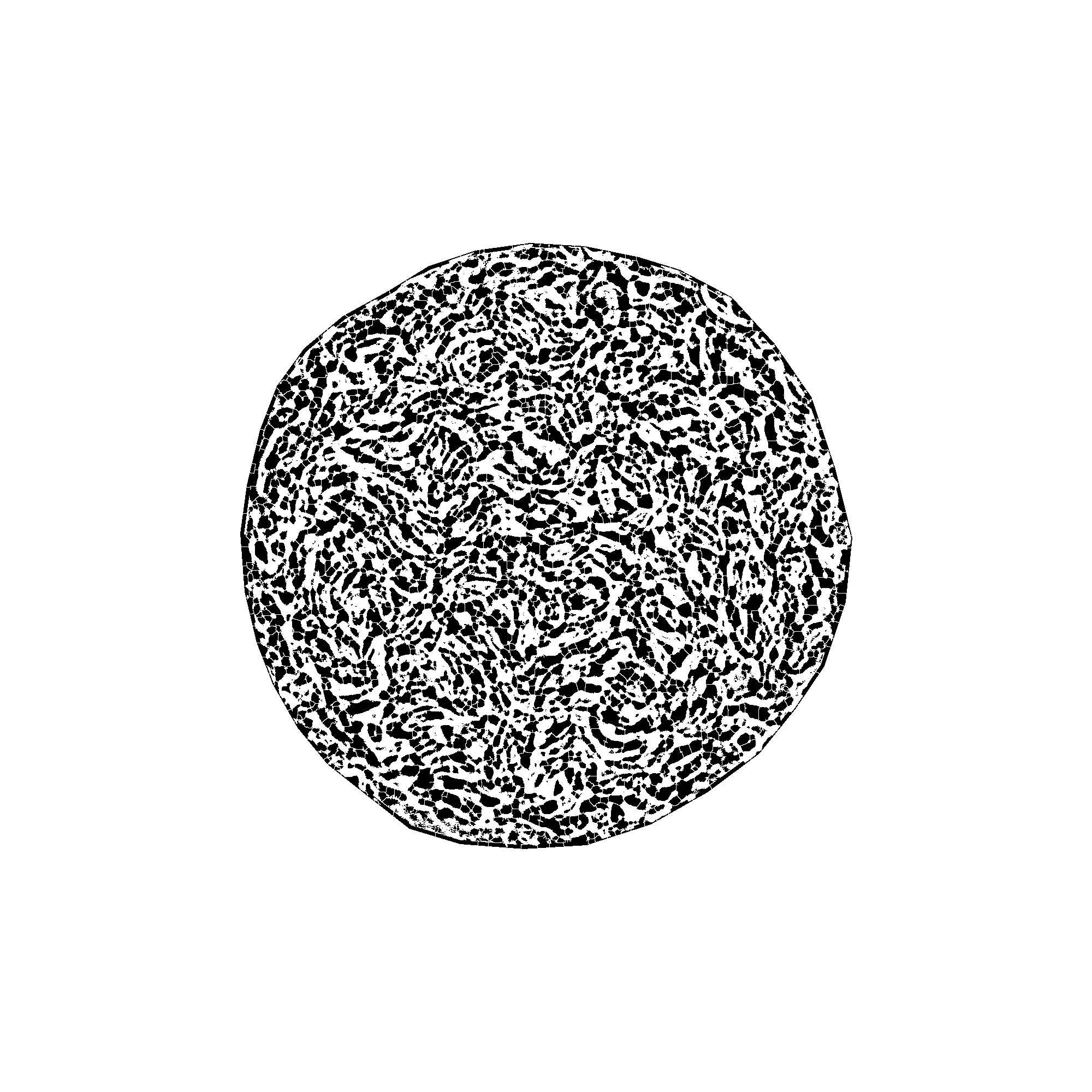

Supplement: S3 Data — (ZIP) [file pone.0234169.s003.zip › Watershed segmentation/BGD26/BGD26-04.tif]

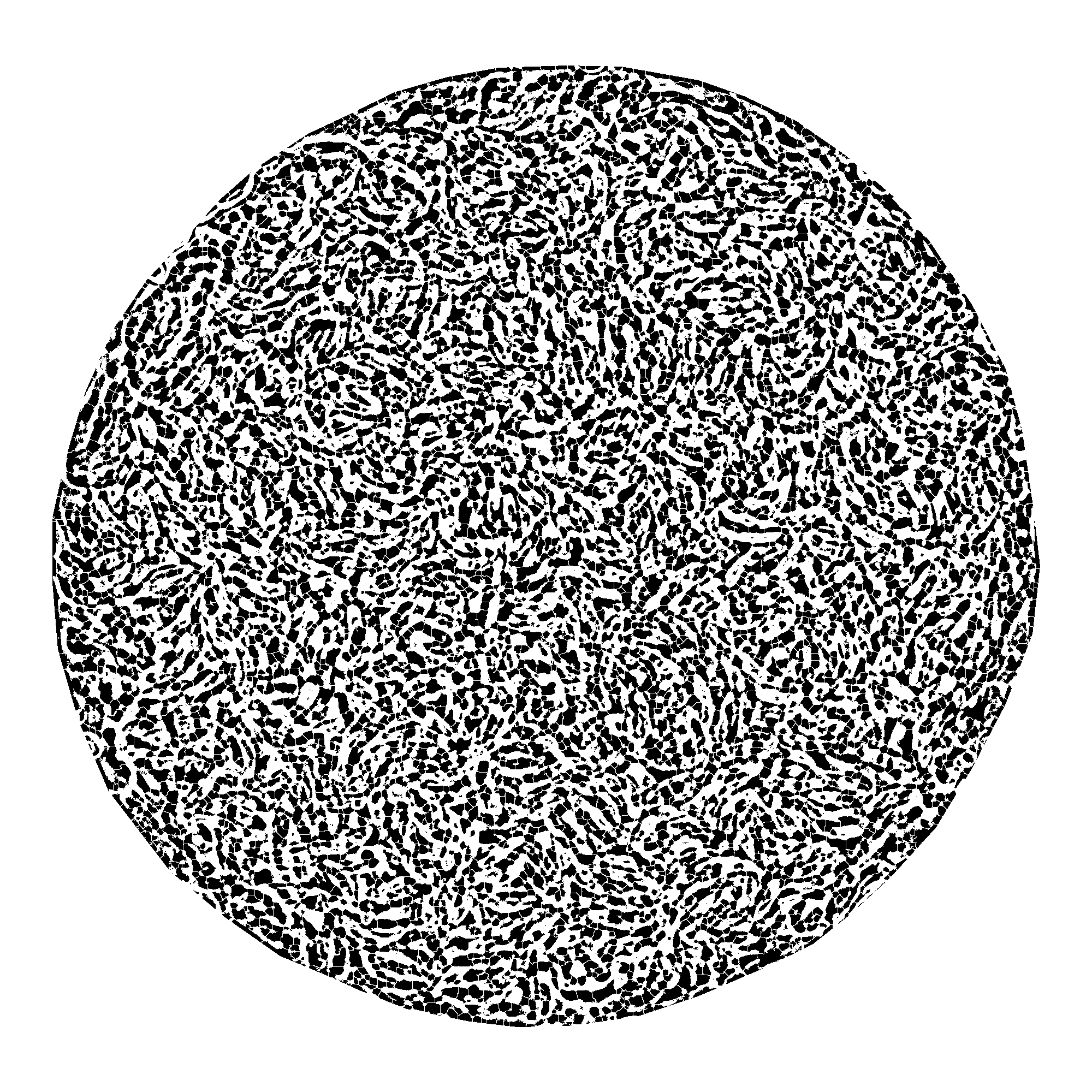

Supplement: S3 Data — (ZIP) [file pone.0234169.s003.zip › Watershed segmentation/BGD26/BGD26-05.tif]

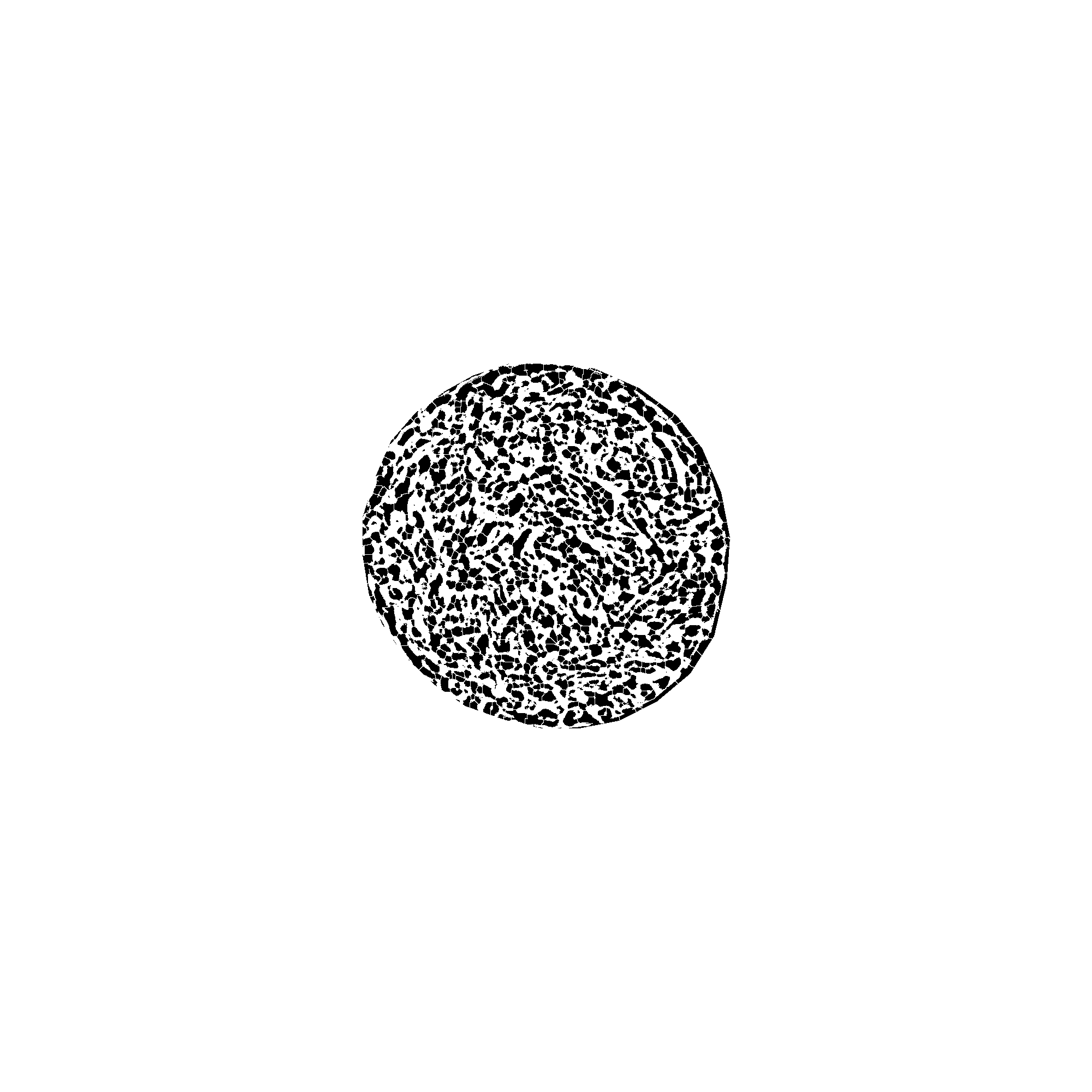

Supplement: S3 Data — (ZIP) [file pone.0234169.s003.zip › Watershed segmentation/BGD26/BGD26-06.tif]

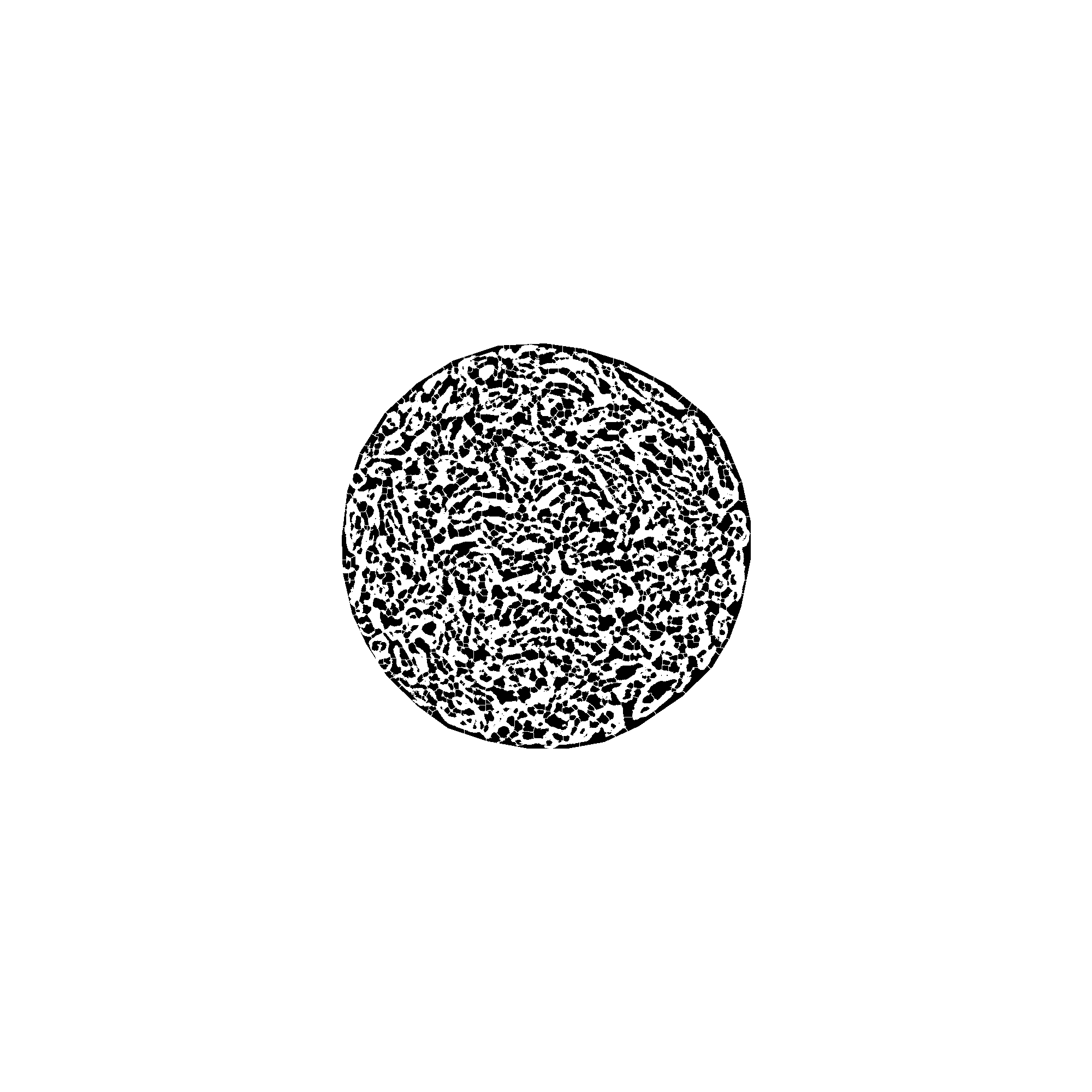

Supplement: S3 Data — (ZIP) [file pone.0234169.s003.zip › Watershed segmentation/ME49 BALBc/ME49 BALBc tp1-01.tif]

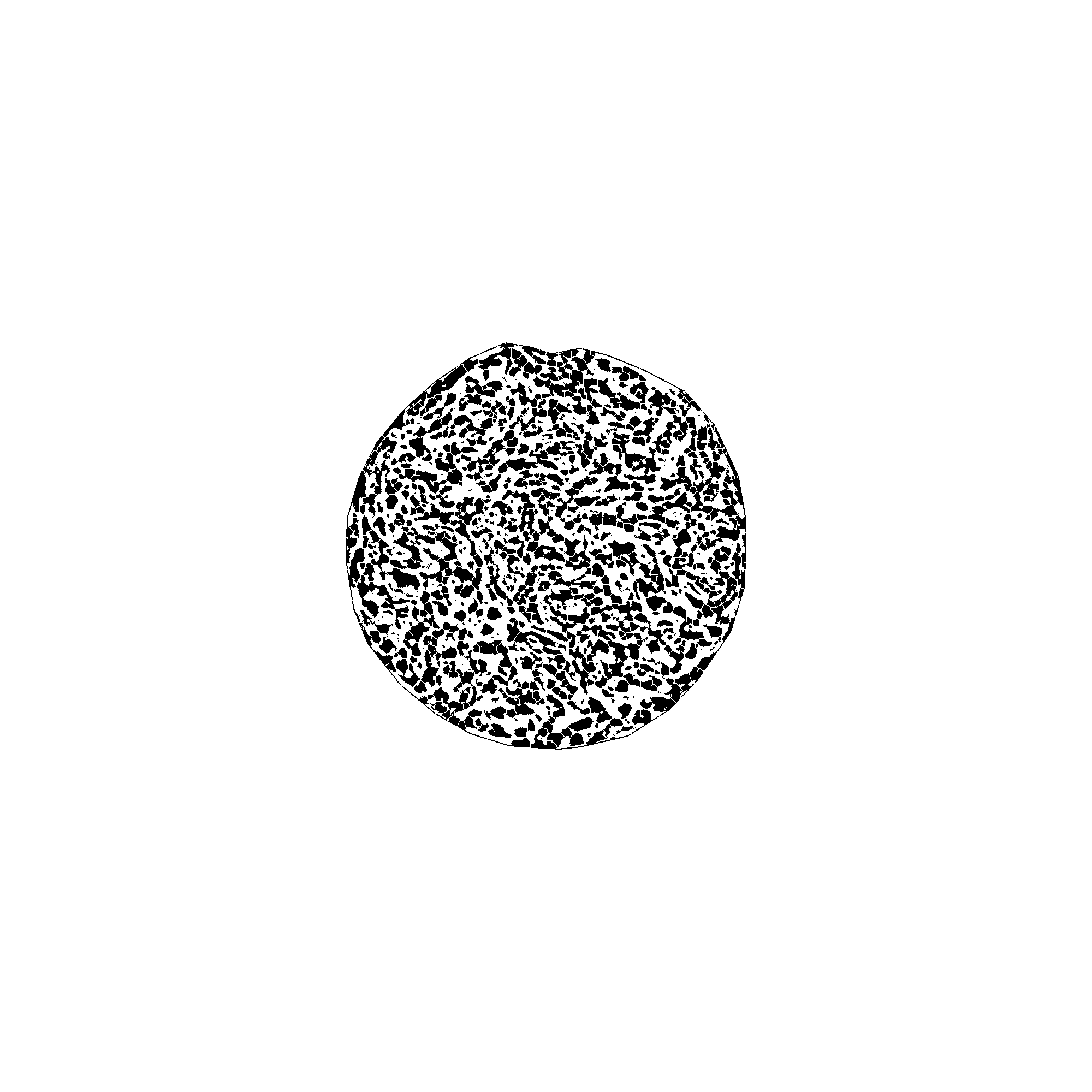

Supplement: S3 Data — (ZIP) [file pone.0234169.s003.zip › Watershed segmentation/ME49 BALBc/ME49 BALBc tp1-02.tif]

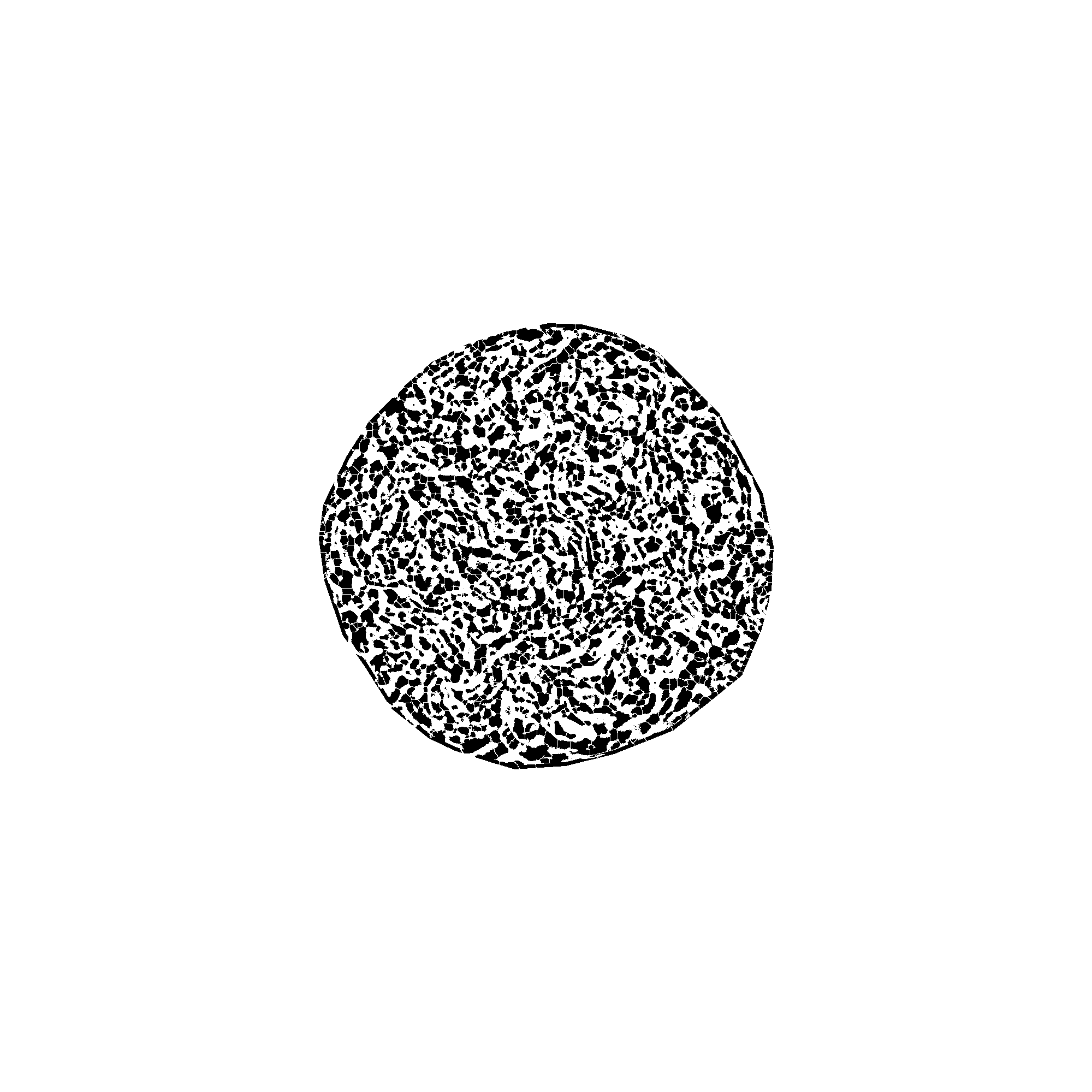

Supplement: S3 Data — (ZIP) [file pone.0234169.s003.zip › Watershed segmentation/ME49 BALBc/ME49 BALBc tp1-03.tif]

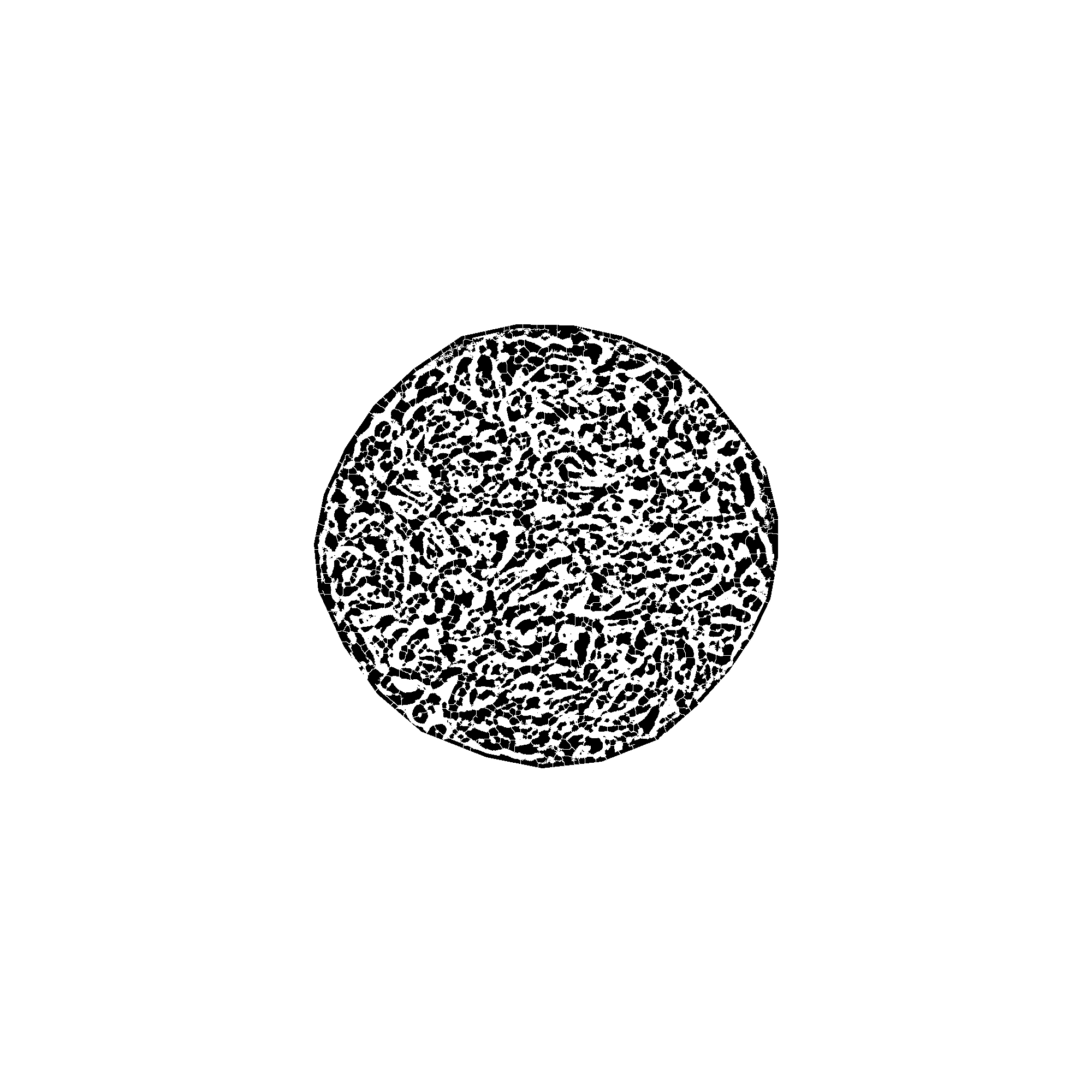

Supplement: S3 Data — (ZIP) [file pone.0234169.s003.zip › Watershed segmentation/ME49 BALBc/ME49 BALBc tp2-01.tif]

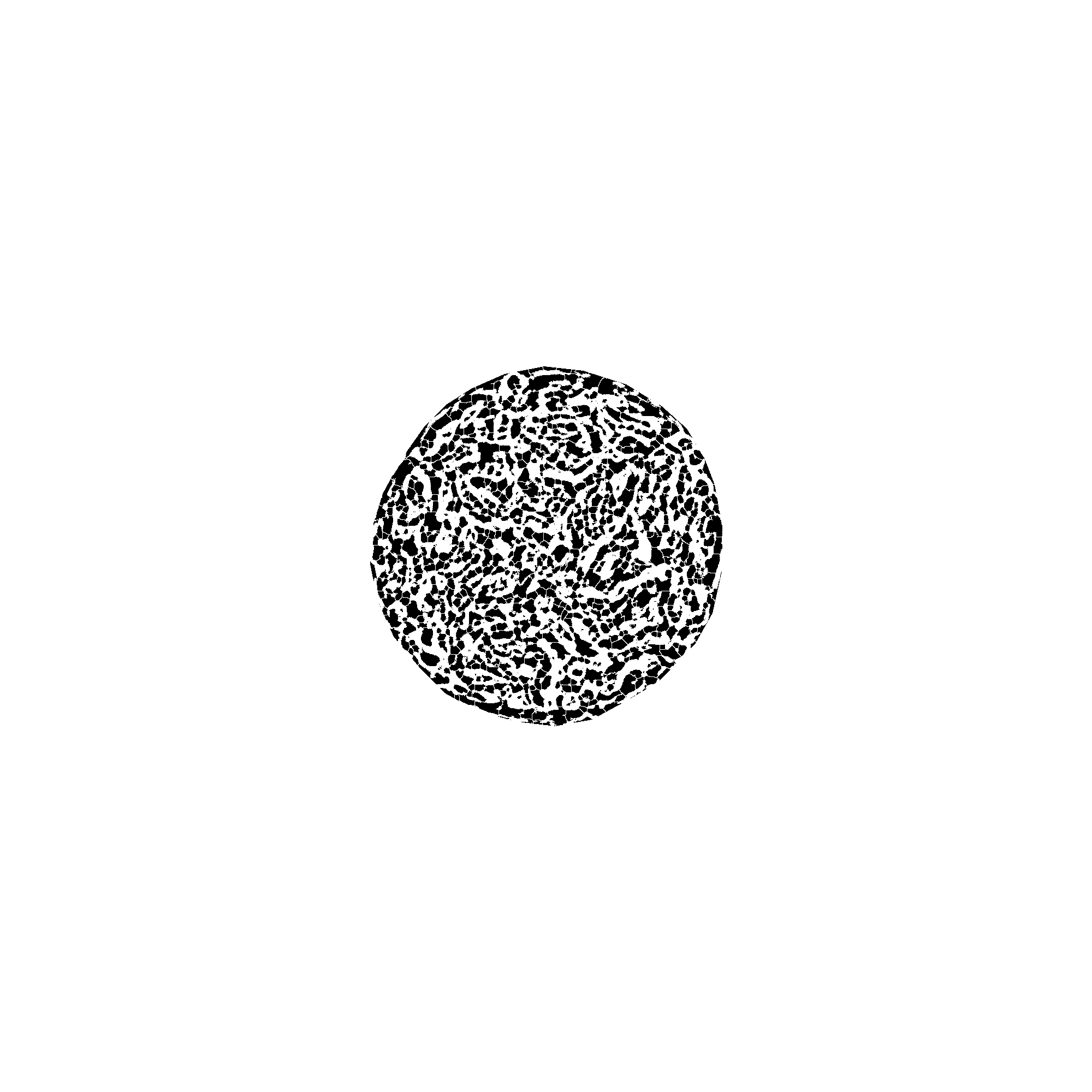

Supplement: S3 Data — (ZIP) [file pone.0234169.s003.zip › Watershed segmentation/ME49 BALBc/ME49 BALBc tp2-02.tif]

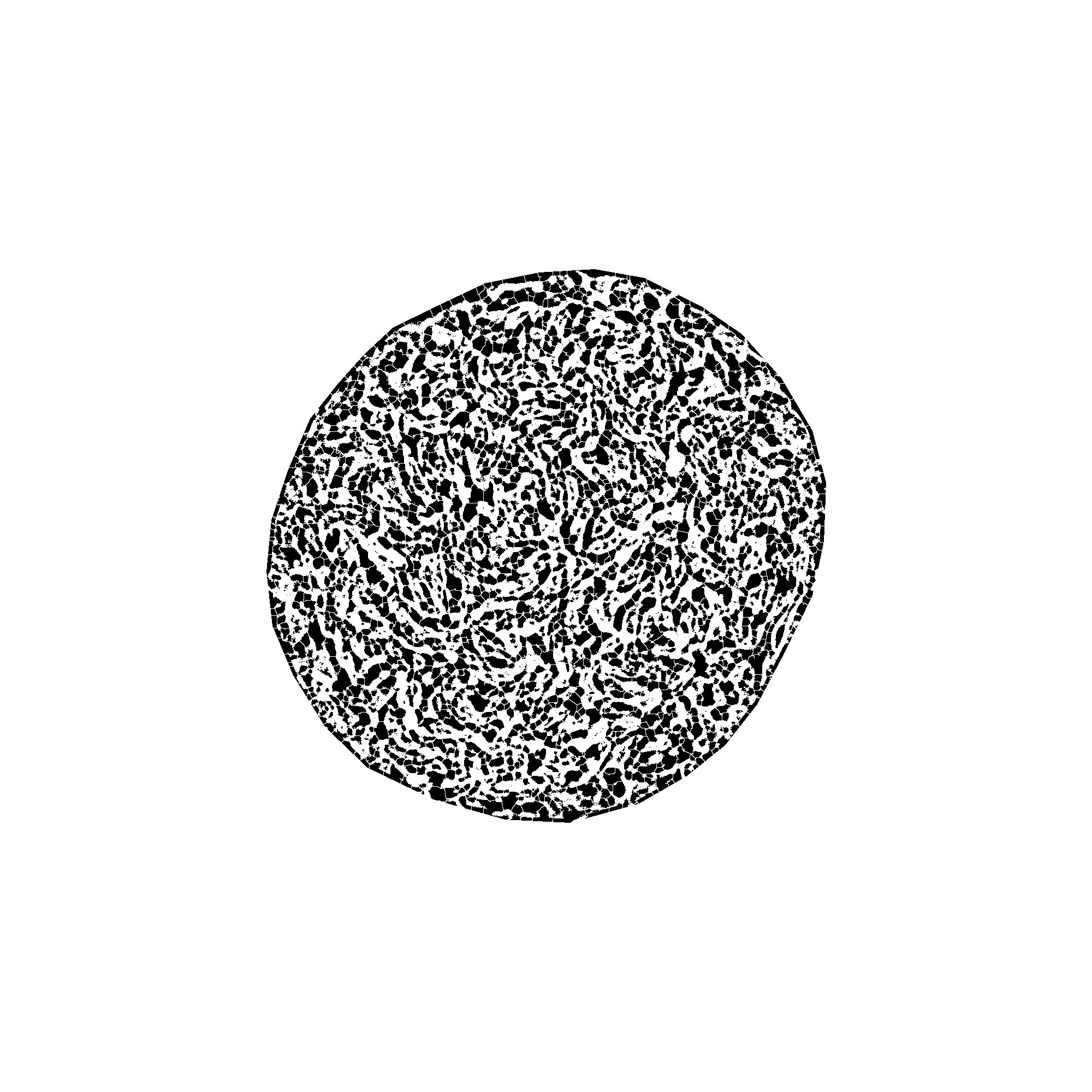

Supplement: S3 Data — (ZIP) [file pone.0234169.s003.zip › Watershed segmentation/ME49 BALBc/ME49 BALBc tp2-03.tif]

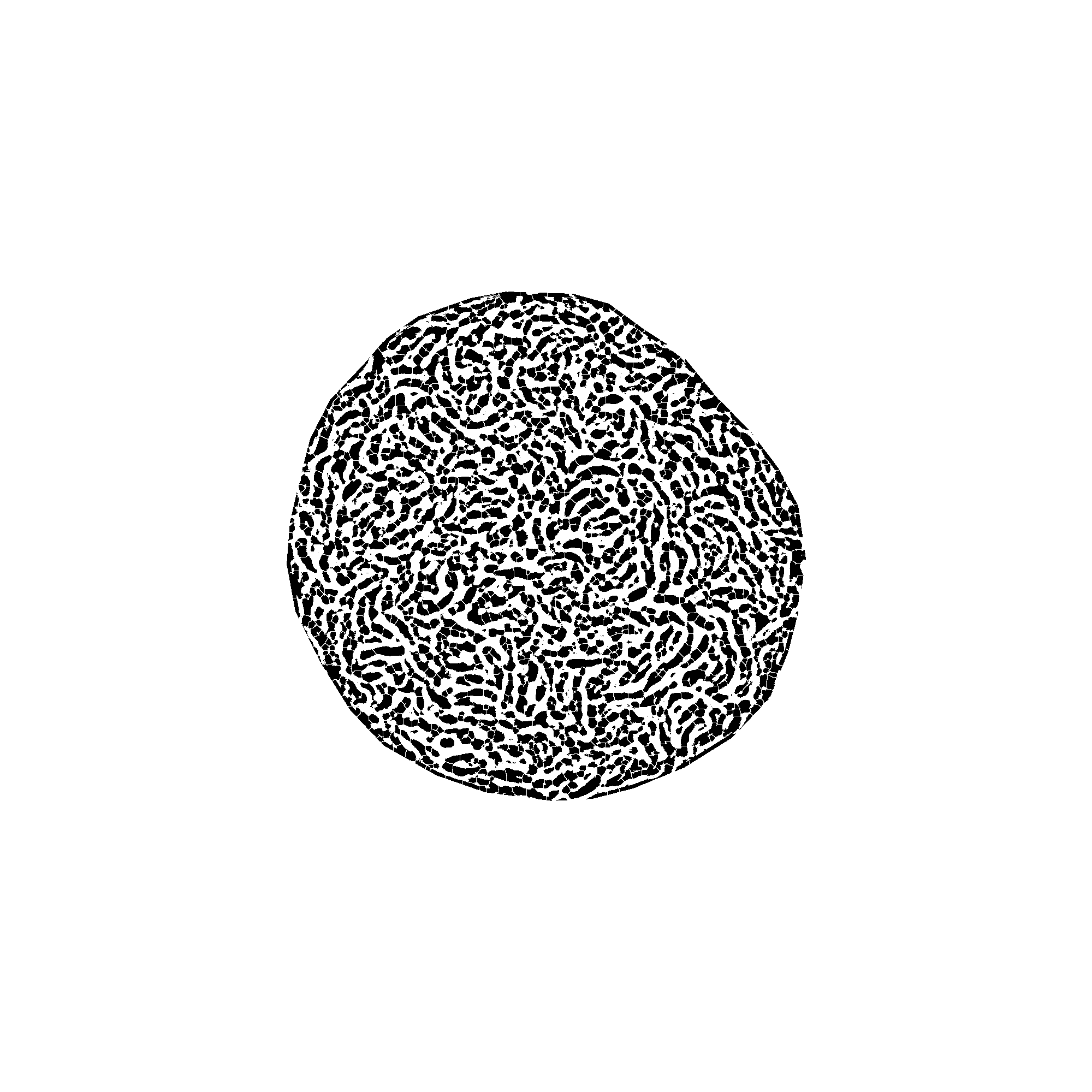

Supplement: S3 Data — (ZIP) [file pone.0234169.s003.zip › Watershed segmentation/ME49 SW/ME49 SW-01.tif]

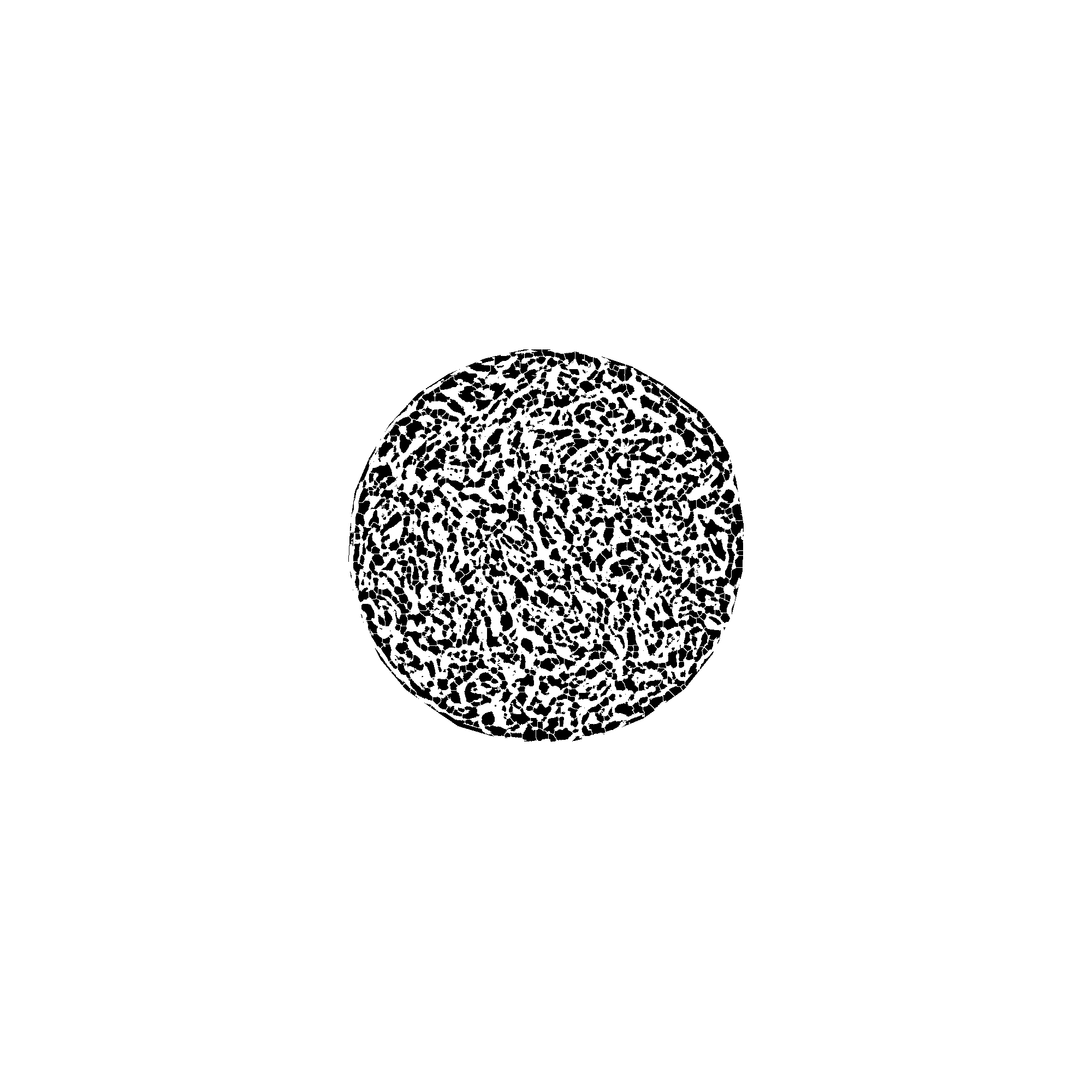

Supplement: S3 Data — (ZIP) [file pone.0234169.s003.zip › Watershed segmentation/ME49 SW/ME49 SW-02.tif]

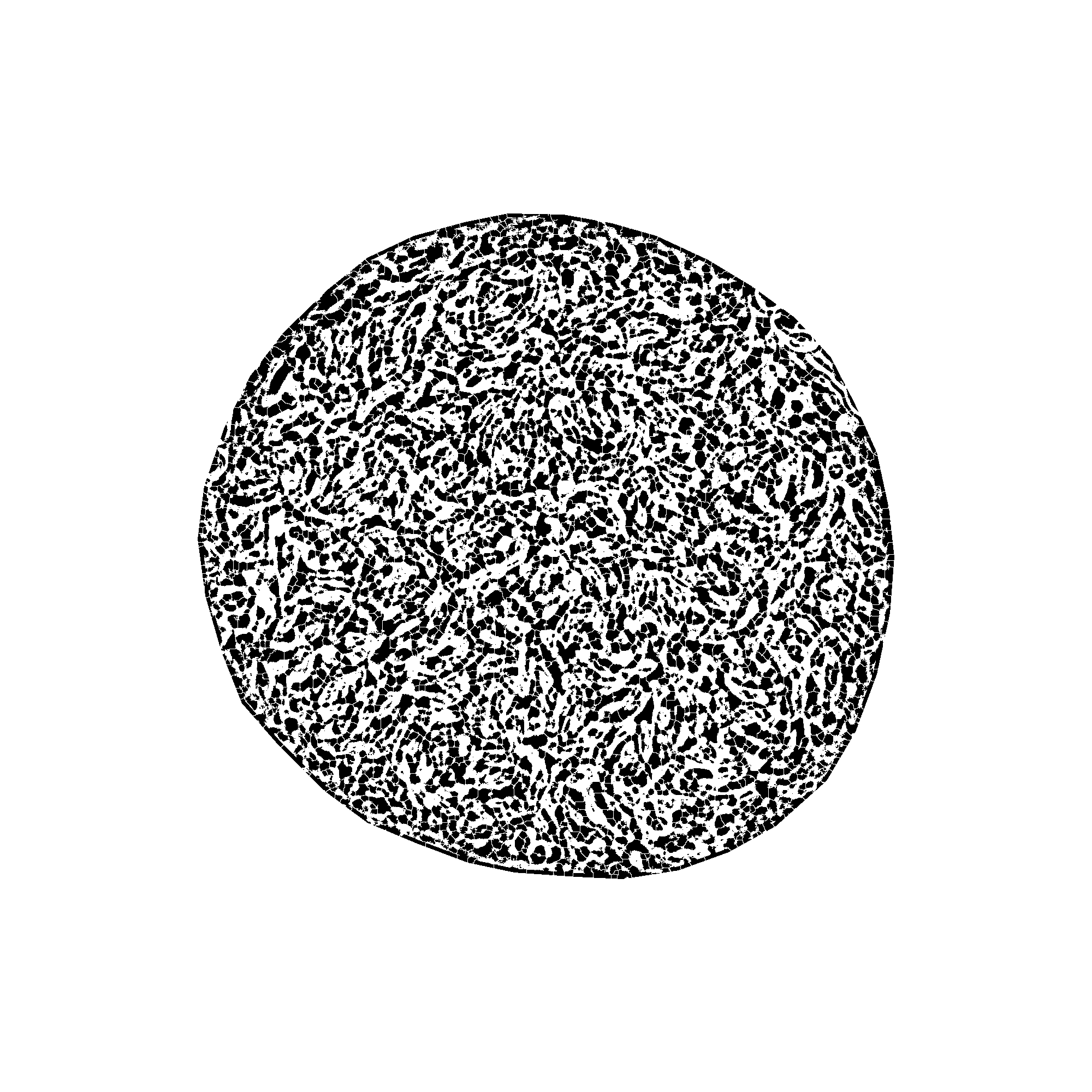

Supplement: S3 Data — (ZIP) [file pone.0234169.s003.zip › Watershed segmentation/ME49 SW/ME49 SW-03.tif]

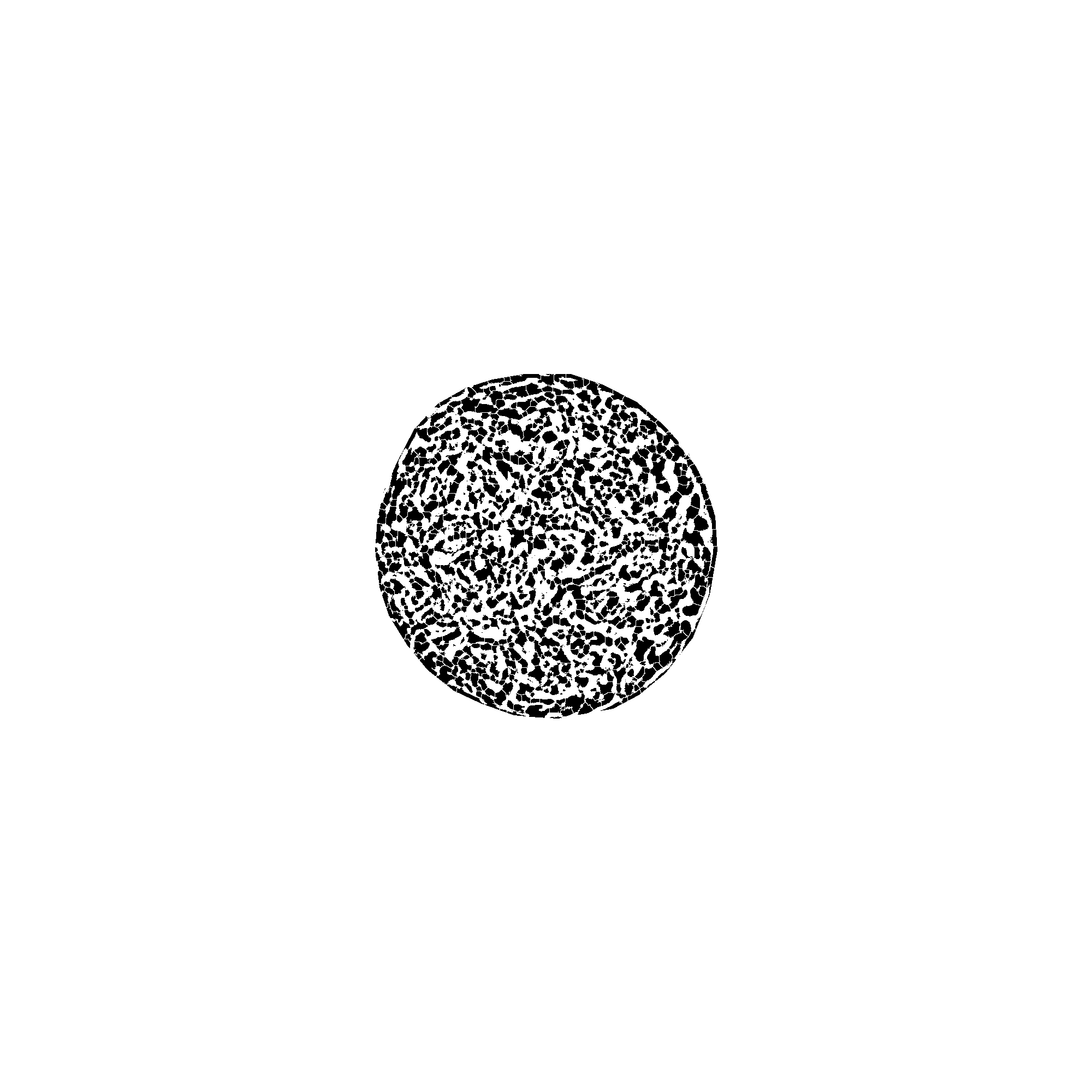

Supplement: S3 Data — (ZIP) [file pone.0234169.s003.zip › Watershed segmentation/ME49 SW/ME49 SW-04.tif]

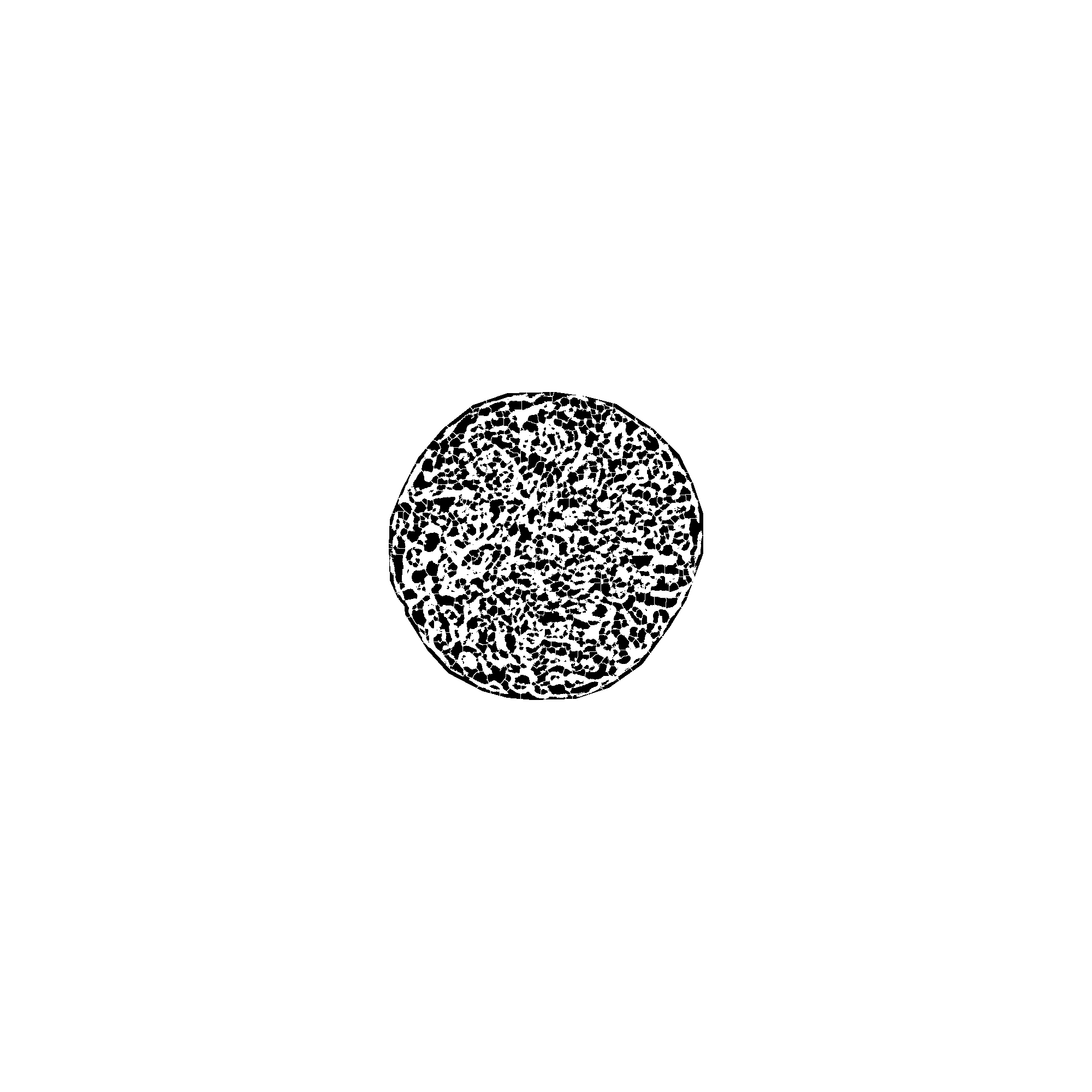

Supplement: S3 Data — (ZIP) [file pone.0234169.s003.zip › Watershed segmentation/ME49 SW/ME49 SW-05.tif]

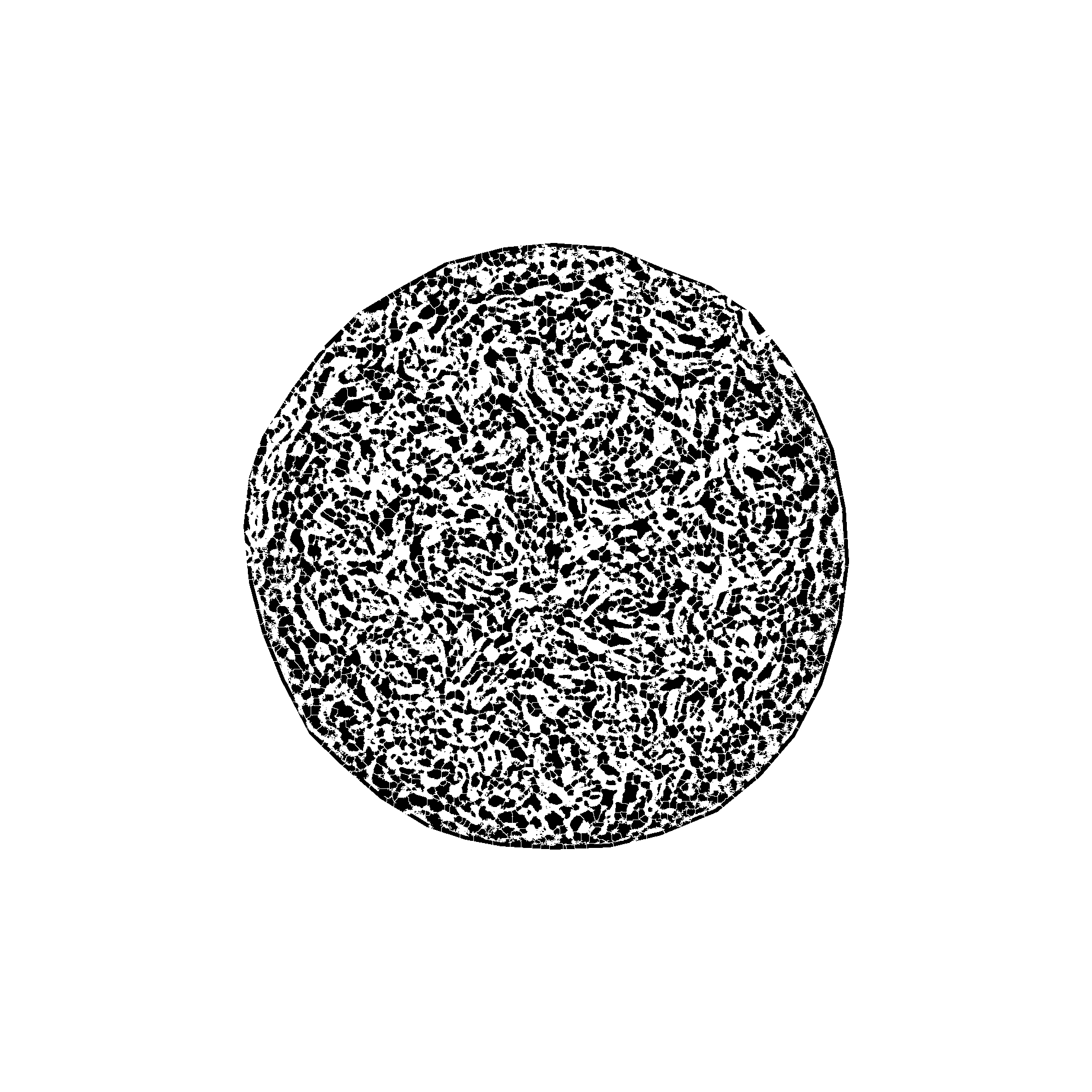

Supplement: S3 Data — (ZIP) [file pone.0234169.s003.zip › Watershed segmentation/ME49 SW/ME49 SW-06.tif]

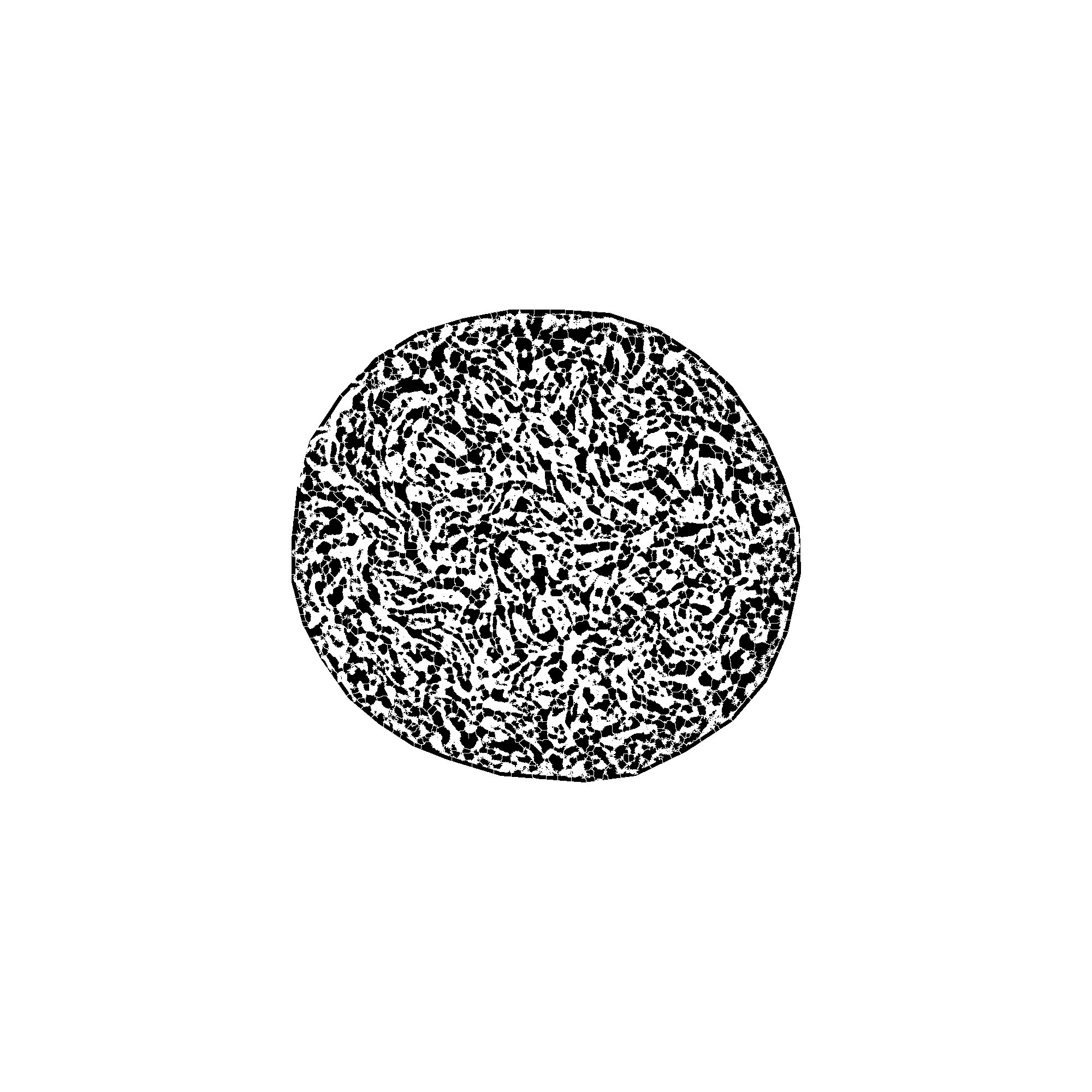

Supplement: S3 Data — (ZIP) [file pone.0234169.s003.zip › Watershed segmentation/ME49 SW/ME49 SW-07.tif]
