# Supplementary material for: Computational image analysis reveals the structural complexity of Toxoplasma gondii tissue cysts
Source: PLoS One. 2020 Aug 18;15(8):e0234169. doi: 10.1371/journal.pone.0234169 (PMC7444489; doi:10.1371/journal.pone.0234169)
Supplement: S1 Appendix — (DOCX) [file pone.0234169.s004.docx]

S1 Appendix

In this section we provide details of the computational image analysis applied to the *T. gondii* cysts. We worked with the 2584 x 1936 px^2^ sized micrographs, taken with the magnification 1000x (oil immersion) using the Axioskop 2 Plus Zeiss phase-contrast microscope and AxioCamMR5 camera. All images were saved in the TIFF format (resolution 150 dpi, bit depth 24).

**Preliminary settings:**

1. Open the ImageJ software (NIH, Bethesda, MD, USA);
2. Initially calibrate the ImageJ software, using the tab: **Analyze/Set scale…**Set unit of length to micrometers (‘um’), Pixel aspect ratio 1.0, and Known distance 1.0. Set distance in pixels to 18.675. We confirmed that in our case the scale was 18.675 pixels per micrometer by multiple measurements of cyst diameters performed by a microscope.
   Make the scale setting ‘Global’;
3. Use the tab **Image/Type/8-bit**  to convert all images into the 8-bit grayscale. Images were previously acquired in gray tones. Save the TIFF files again.

**Cyst circularity calculation / transfer to the black background:**

The following operations were applied:

1. A cyst wall is precisely delineated using the polygon tool macro preinstalled in ImageJ:
   *
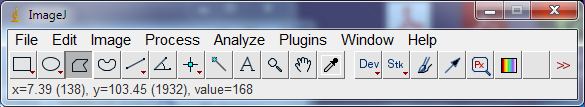
* .
2. Use the tab **Analyze/Set Measurements…** to choose the parameters of interest for automatic size, shape, and gray level distribution analysis. Define the desired number of decimal places (we used six decimal places). We kept the ticks on the following: Area, Centroid, Perimeter, Fit ellipse, Shape descriptors, Feret’s diameter. *Circularity* is one of the four parameters provided as Shape descriptors. Due to the very high *Circularity*, and an excellent agreement of the Area, Perimeter, and Feret’s diameter data with the cyst diameter measured using a microscope, a comparison of other recorded parameters did not yield any additional insight. It is kept on record in our laboratory, for internal use, but not used in the manuscript.
3. Click on **Ctrl+M**, or use **Analyze/Measure…** tab to record the data in a table. Leave the table open during the analysis of a current cyst group. When cyst analysis is completed save the table using the **File/Save As…** option (or **Ctrl+S**), as a CSV file.
4. Click on **Ctrl+D**, or use **Edit/Draw** tab to invasively draw the outline and record this image as a separate TIFF file, for further use. Use **Edit/Options/Colors…** to define an outline color and **Edit/Options/Line Width…** to define a desired line width.
5. Click on **Ctrl+C**, or use **Edit/Copy** tab to copy the interior of the outline, i.e., to copy pixels belonging to a cyst to the clipboard. Click on **Ctrl+N**, or use **File/New/Image…** tab to create an appropriately sized new image of a cyst without the surroundings. Define image Name, set Type: 8-bit, Fill with: Black, Slices: 1, Width/Height: 1700 x 1700. Paste the clipboard data onto a new image using **Ctrl+V**, or use **Edit/Paste** tab.

**Particle analysis:**

1. Perform histogram equalization using **Process/Enhance Contrast…** tab. Place tick on **Equalize Histogram…** option and click **OK**. (Saturated pixels are not used with this option.)
2. Use the tab **Image/Adjust/Auto Local Threshold…** to perform local auto thresholding. Set Method to **Bernsen**, Radius: **7**, Parameter 1: 0 (default), Parameter 2: 0 (default), White objects on black background: Yes (tick).
3. Use **Process/Binary/Make Binary** to convert the thresholded images to binary. Use **Process/Binary/Watershed** to perform watershed segmentation, enabling the assessment of the packing density (***PD***).
4. Open the **Analyze/Analyze Particles…** tab. Set the size range to Size (μm^2): **0.04 – 144.00**. Choose only **Summarize** option (tick). Choose Show: **Masks**.

**Fractal analysis:**

1. Starting from the images described in bullet point 8, take 450 x 450 px^2^ sized cutouts close to the cyst center to acquire equally sized visual texture images for all analyzed cysts. Use the **Image/Adjust/Threshold…** option (**Ctrl+Shift+T**) to obtain the contours for fractal analysis. Otsu threshold will be automatically applied. Adjust the lower histogram value to keep only the narrow interval of gray tones including the Otsu threshold. Choose **Apply**, and then use **Process/Binary/Make Binary**.
2. FracLac plugin can be found at <https://imagej.nih.gov/ij/plugins/fraclac/fraclac.html>. It is installed simply by copying the files into the directory of interest (as explained at the link). Restart the ImageJ upon installation. Open the FracLac plugin user interface.
3. Click on the **BC icon** at the left, in the top row, to adjust the settings for the standard fractal analysis. Use the Regular mode. Set **Image Type** options to Use Binary, and Lock White Background. In the **Grid Design** section tick on the **‘random’**, for random grid positioning, and set the number of analysis grids to twelve. Choose **Scaled Series**: set numerator/denominator to 7 over 8. **Sizes**: choose to define min size, as well as max size, in pixels. Set the min size to 16, and the max size to 320. A total of 23 grid sizes for analysis is obtained.
4. **Graphics Options**: tick the boxes ‘regression’ and ‘lacunarity’ to obtain and check on the plots of regression lines used in calculating the fractal dimension and lacunarity. Also tick ‘draw grids’ to see the actual grid positioning for various grid sizes. **Files**: tick the box ‘results’ to save the calculation results to files. Otherwise, tables are given on screen and have to be saved using the **File/Save As…** option (or **Ctrl+S**).
5. Confirm the settings by clicking on **OK**, and further use only the **Scan** button (bottom row on the left) to scan all of the images using the settings adjusted as explained above. Twelve sets of data per cyst were obtained with different random grids. All data relevant to fractal analysis was automatically recorded in tables to be used for further comparisons.
